# Tibetan Medicine Duoxuekang Capsule Ameliorates High-Altitude Polycythemia Accompanied by Brain Injury

**DOI:** 10.3389/fphar.2021.680636

**Published:** 2021-05-11

**Authors:** Ke Chen, Ning Li, Fangfang Fan, ZangJia Geng, Kehui Zhao, Jing Wang, Yi Zhang, Ce Tang, Xiaobo Wang, Xianli Meng

**Affiliations:** ^1^ School of Pharmacy, Chengdu University of Traditional Chinese Medicine, Chengdu, China; ^2^ School of Ethnic Medicine, Chengdu University of Traditional Chinese Medicine, Chengdu, China; ^3^ School of Pharmacy, Southwest Minzu University, Chengdu, China; ^4^ School of Management, Chengdu University of Traditional Chinese Medicine, Chengdu, China; ^5^ Ethnic Medicine Academic Heritage Innovation Research Center, Chengdu University of Traditional Chinese Medicine, Chengdu, China; ^6^ NMPA Key Laboratory for Quality Evaluation of Traditional Chinese Medicine (Traditional Chinese Patent Medicine), Chengdu University of Traditional Chinese Medicine, Chengdu, China; ^7^ State Key Laboratory of Southwestern Chinese Medicine Resources, Innovative Institute of Chinese Medicine and Pharmacy, Chengdu University of Traditional Chinese Medicine, Chengdu, China

**Keywords:** Tibetan medicine, Duoxuekang capsule, high-altitude polycythemia accompanied by brain injury, MAPK signaling pathway, RAS signaling pathway

## Abstract

**Objective:** Duoxuekang (DXK) capsule is an empirical prescription for Tibetan medicine in the treatment of hypobaric hypoxia (HH)-induced brain injury in the plateau. This study aimed to investigate the protective effects and underlying molecular mechanisms of DXK on HH-induced brain injury.

**Methods:** UPLC–Q-TOF/MS was performed for chemical composition analysis of DXK. The anti-hypoxia and anti-fatigue effects of DXK were evaluated by the normobaric hypoxia test, sodium nitrite toxicosis test, and weight-loaded swimming test in mice. Simultaneously, SD rats were used for the chronic hypobaric hypoxia (CHH) test. RBC, HGB, HCT, and the whole blood viscosity were evaluated. The activities of SOD and MDA in the brain, and EPO and LDH levels in the kidney were detected using ELISA. H&E staining was employed to observe the pathological morphology in the hippocampus and cortex of rats. Furthermore, immunofluorescence and Western blot were carried out to detect the protein expressions of Mapk10, RASGRF1, RASA3, Ras, and IGF-IR in the brain of rats. Besides, BALB/c mice were used for acute hypobaric hypoxia (AHH) test, and Western blot was employed to detect the protein expression of p-ERK/ERK, p-JNK/JNK, and p-p38/p38 in the cerebral cortex of mice.

**Results:** 23 different chemical compositions of DXK were identified by UPLC–Q-TOF/MS. The anti-hypoxia test verified that DXK can prolong the survival time of mice. The anti-fatigue test confirmed that DXK can prolong the swimming time of mice, decrease the level of LDH, and increase the hepatic glycogen level. Synchronously, DXK can decrease the levels of RBC, HGB, HCT, and the whole blood viscosity under the CHH condition. Besides, DXK can ameliorate CHH-induced brain injury, decrease the levels of EPO and LDH in the kidney, reduce MDA, and increase SOD in the hippocampus. Furthermore, DXK can converse HH-induced marked increase of Mapk10, RASGRF1, and RASA3, and decrease of Ras and IGF-IR. In addition, DXK can suppress the ratio of p-ERK/ERK, p-JNK/JNK, and p-p38/p38 under the HH condition.

**Conclusion:** Together, the cerebral protection elicited by DXK was due to the decrease of hematological index, suppressing EPO, by affecting the MAPK signaling pathway in oxidative damage, and regulating the RAS signaling pathway.

## Introduction

There are about 140 million residents in the plateau section all over the world (Boucly et al., 2017). Hypobaric hypoxia (HH) environment at high altitude is one of the main factors affecting human life activities (West, 2016). Furthermore, with the development of society and economy, thousands of people are climbing to high-altitude areas for reasons such as work or tourism (Luan et al., 2019). People at high altitudes (≥2500 m) would suffer from acute mountain sickness (AMS) or chronic mountain sickness (CMS), manifested by headache, insomnia, and dyspnea (Ma et al., 2020). Besides, AMS simultaneously causes nausea, vomiting, and dizziness, and can even be life threatening, while CMS can result in dyspepsia and high risk of thrombosis, affecting more than 80 million people worldwide (Gazal et al., 2019). Some evidences show that the prevalence of CMS in high-altitude populations varies from 1.2 to 33% (Bao et al., 2017) and is about 20% among Andean highlanders (Yao et al., 2018). Synchronously, CMS is characterized by high-altitude polycythemia (HAPC) and high-altitude pulmonary hypertension (Gao et al., 2020). Furthermore, patients with HAPC experience venectasia, attention-deficit disorder, and lapse of memory (Liu et al., 2018).

It was reported that the morbidity of HAPC was around 5–18% in the Qinghai–Tibet Plateau (León-Velarde et al., 2005), and its prevalence increased with the elevation of altitude (Fan et al., 2018). HAPC accompanied by brain injury was a common clinical symptom, which seriously damaged public health (Deng et al., 2020). It was confirmed that the expression of EPO can be increased after HH exposure (Yang et al., 2021), which can lead to increased erythropoiesis (Kasperska and Zembron-Lacny, 2020). Excessive erythrocytosis results in an increase in blood viscosity which can impair blood flow (Ogunshola et al., 2006). With the increase of blood viscosity, the flow rate of blood in the body slows down, leading to decreased blood perfusion in the brain tissue (Frietsch et al., 2017). It was confirmed that the brain tissue of HAPC patients was in a hypoxic and ischemic state, which was prone to intracranial ischemia, infarction, and hemorrhage (Bao et al., 2019). It had been confirmed that the activation of the MAPK signaling pathway led to brain injury after HH exposure (Wang et al., 2018). Furthermore, it was reported that the Ras/Raf/ERK pathway was critical for neuroprotection and apoptosis suppression during hypoxia insult (Xu et al., 2016).

Duoxuekang capsule (DXK, 
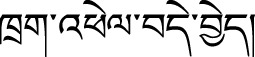
), composed of *Phyllanthus emblica* L. (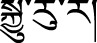, Ju Rure), *Rhodiola crenulata* (Hook. f. et Thoms.), H. Ohba (
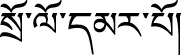
, Suoluo Mabu), *Hippophae rhamnoides* L. (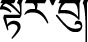, Daerbu), and *Zingiber officinale* Rosc. (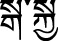, Gajia) (Wang et al., 2017), was derived from a secret recipe owned by Cuoru-Cailang, a very well-known Tibetan medicine master. In the previous studies, we have confirmed that HIF-1 alpha (HIF-1α) but not HIF-1 beta will undergo significant changes during hypoxia stimulation, and *Rhodiola crenulata* can regulate the expression of HIF-1α to exert an anti-hypoxia cerebral protective role (Wang et al., 2019). In addition, *Rhodiola crenulata* can improve HH-induced brain injury by inhibiting the apoptosis of the hippocampus and maintaining the morphology and structure of mitochondria (Hou et al., 2018). As a frequently used prescription of Tibetan medicine, DXK is effective in the treatment of HAPC. Clinical investigations found that DXK can enrich brain–blood perfusion of patients with HAPC through enhancing the oxyhemoglobin saturation and heart rate (Ga et al., 2019; Li et al., 2020). Simultaneously, we also confirmed that DXK can increase the number of collagen and elastic fibers in AHH-induced brain injury *via* inhibiting oxidative stress injury (Li et al., 2020). In this study, we first analyzed the chemical compositions of DXK by UPLC–Q-TOF/MS and investigated whether its underlying molecular mechanisms on HAPC accompanied by brain injury were related to MAPK and RAS signaling pathways.

## Materials and Methods

### Reagents and Chemicals

Duoxuekang capsule (DXK, No. 160816) was provided by the research laboratory of School of Ethnic Medicine, Chengdu University of Traditional Chinese Medicine (Chengdu, China). Nuodikang capsules (NDK, No. Z10980020) were produced by Tibet Rhodiola Pharmaceutical Holding Co., Ltd. (Tibet, China). Hongjingtian oral liquid (HOL, No. Guoyaozhunzi B20070002) was provided by Tibet Tibetan Medicine Group Co. LTD (Tibet, China). Superoxide dismutase (SOD, No. A001-3-2), lactic dehydrogenase (LDH, No. A020-2-2), erythropoietin (EPO, No. H051), malondialdehyde (MDA, No. A003-1-2), hepatic glycogen (No. A043-1-1), and total protein extraction kits (W034-1-1) were purchased from Nanjing Jiancheng Bioengineering Institute (Nanjing, China). Bicinchoninic acid (BCA) Protein Quantitative Kit (70-PQ0012) was provided by Multi Sciences (Lianke) Biotech, Co., Ltd. (Hangzhou, China). Anti-Mapk10 (ET1612-68), anti-Ras (ER40115), and anti–IGF-IR antibodies (ER63734) were provided by Hangzhou Hua’an Biotechnology Co. Ltd. (Hangzhou, China). Anti-RASGRF1 (bs-3560) and anti-RASA3 antibodies (bs-595) were purchased from Bioss Biological Technology, Ltd. (Beijing, China). Cy3-conjugated goat anti-rabbit IgG (GB21303), bull serum albumin (BSA, G5001), and ethylene diamine tetraacetic acid (EDTA) antigen retrieval solution (PH8.0, G1206) were provided by Wuhan Servicebio Technology Co., Ltd. (Wuhan, China). RASGRF1 (A6964) was purchased from ABclonal Biotechnology Co, Ltd. (Wuhan, China). Antibodies against p-ERK (#9101), ERK (#9102), p-JNK (#9251), JNK (#9252), p-p38 (#4511), p38 (#8690), β-actin (#8457), and anti-rabbit immunoglobulin G secondary antibodies (#7074) were provided by Cell Signaling Technology, Inc. (Danvers, MA, United States). Sodium dodecyl sulfate–polyacrylamide gel electrophoresis (SDS-PAGE, AR0131) and polyvinylidene fluoride (0.45 μm, PVDF, AR0136-04) were provided by Boster Biological Technology Co., Ltd. (Wuhan, China). Ultrasignal ECL chemiluminescent solution (4AW011-200) was purchased from Beijing 4A Biotech Co., Ltd. (Beijing, China). Methanol and acetonitrile for UPLC were purchased from J. T. Baker Inc (Phillipsburg, NJ, United States). Leucine enkephalin and formic acid were supplied by Sigma-Aldrich (St Louis, MO, United States).

### UPLC–Q-TOF-MS Analysis

The chemical compositions of DXK were identified by UPLC–Q-TOF/MS according to the previous study (Zhang et al., 2020). The chromatographic method was achieved adopting Acquity UPLCR BEH C18 (100 mm × 2.1 mm × 1.7 μm) as a stationary phase and 0.1% formic acid (A)/acetonitrile with 0.1% formic acid (B) as a mobile phase with gradient elution at a constant flow rate of 0.4 ml/min. The elution order was as follows: maintained with 2% B in 3 min, linear gradient from 2% B to 5% B in 2 min, 5% B to 8% B in 1 min, 8% B to 9% B in 5 min, 9% B to 12% B in 6 min, 12% B to 21% B in 9 min, 21% B to 25% B in 2 min, and 25% B to 35% B in 2 min. The column temperature was carried out at 40°C, and the injection volume was 1 μl. The Waters SYNAPT G2 HDMS system was used for mass spectrometry. Nitrogen is used as atomizing hole gas, with source temperature, 150°C; cone gas flow, 50 l/h; desolvation temperature, 450°C; desolvation gas flow, 800 l/h; sampling cone, 40 V; extraction cone, 4 V; capillary voltage, 2.5 kV (negative mode); scan time, 0.2 s; inter-scan time, 0.02 s; mass-to-charge ratio, m/z 100–1200 Da; and lock mass (leucine enkephalin), m/z 554.2615 [M-H] (negative-ion mode). The data were analyzed using MassLynx V4.1 software (Waters).

### Animals

All animals were provided by Experimental Animal Institute of Sichuan People’s Hospital (License number: SCXK (Chuan) 2015–30) and were tested after 7 days of adaptive feeding in a well-ventilated environment under a 12-h dark/light cycle and dark cycle at 23 ± 2°C and humidity of 60 ± 5% in the animal room of Plateau Disease Laboratory, School of Ethnic Medicine, Chengdu University of Traditional Chinese Medicine.

### Hypoxia Tolerance Test in Mice

Eighty male Kunming species mice (20 ± 2 g, 6–8 weeks) were randomly divided into five groups: control group, NDK group (0.28 g/kg), DXK low-, medium-, and high-dose groups (DXK-L, 0.9 g/kg; DXK-M, 1.8 g/kg; DXK-H, 3.6 g/kg). Each group was randomly divided into two groups with eight mice in each group, and the mice were administered continuously for 30 days. After the last intragastric administration, each mouse was allowed to rest for 1 h. For the normobaric hypoxia test, the mice were placed in a 250-ml airtight container containing 10 g of medical soda lime (Xie et al., 2020). For the sodium nitrite toxicosis test, the mice were intraperitoneally injected with 2% (m/v) sodium nitrite solution at 20 ml/kg (Yang et al., 2019). The survival time was recorded until the disappearance of abdominal breathing.

### Weight-Loaded Swimming Test

Forty male Kunming species mice (20 ± 2 g, 6–8 weeks) were randomly divided into five groups: control group, NDK group (0.28 g/kg), DXK low-, medium-, and high-dose groups (DXK-L, 0.9 g/kg; DXK-M, 1.8 g/kg; DXK-H, 3.6 g/kg). After the last intragastric administration, the mice were loaded with a lead wire of 4% of bodyweight attached to their tails. Then, the mice were gently placed into a plastic pool to a depth of 30 cm filled with water at 25 ± 0.5°C for swimming. Each mouse was individually placed in the pool to reduce interference. The exhausted swimming time was recorded when the mice failed to return to the surface to breathe within a period of 10 s (Hou et al., 2020; Li et al., 2020).

### Chronic Hypobaric Hypoxia Experiment

Thirty-six male Sprague–Dawley rats (200 ± 20 g, 2–3 months) were randomly divided into six groups: control group, model group, NDK group (0.14 g/kg), DXK-L group (0.45 g/kg), DXK-M group (0.9 g/kg), and DXK-H group (1.8 g/kg), with six rats in each group. The control group was served as normoxia kept at normal atmospheric pressure, and other groups were exposed continuously for 40 days to a simulated high altitude of 5000 m in an animal hypobaric and hypoxic chamber (FLYDWC50-II C, Avic Guizhou Fenglei Aviation Armament Co., Ltd, China). The rate of ascents to altitude was maintained at 300 m/min. The chamber was brought down to sea level at every day interval for 30 min for intragastric administration and replenishment of food and water (Maiti et al., 2008).

### Analysis of Hematological Parameters of Rats

On the 40th day, the rats were intraperitoneally anesthetized with 200 mg/kg pentobarbital sodium, and the blood was taken from the abdominal aorta using one-time anticoagulant negative pressure blood collection tubes. The blood samples were kept statically at room temperature for 20 min; then, red blood cell count (RBC), hemoglobin (HGB), red blood cells deposited (HCT), and the whole blood viscosity were measured by an automatic three-group blood analyzer (TEK- MINI, Jiangxi Tecom Technology Co., LTD, Jiangxi, China). Additionally, the whole blood viscosity was measured at four shear rates of 1/S, 5/S, 50/S, and 200/S (Nader et al., 2018) by the full-automatic hemorheology testing instrument (SA-6000, Beijing Secco Sid Technology Co., LTD, Beijing, China). The diagnostic criterion of HAPC was as follows: HGB ≥ 210 g/l (Li et al., 2014).

### Biochemical Analysis of Rats

After weight-loaded swimming test, the serum LDH and hepatic glycogen were determined according to the manufacturer’s instructions. After removal of the brain and kidney, the samples of rat subject to CHH insult were washed in 4°C physiological saline and preserved in a refrigerator at −80°C. Then, the 10% (w/v) sample homogenates were centrifuged at 3500 rpm for 10 min (Liu et al., 2019). The supernatant was harvested to determine the levels of SOD and MDA (brain), as well as EPO and LDH (kidney). The absorbance at 450 nm (SOD and LDH), 532 nm (MDA), and 620 nm (EPO) was determined by a microplate reader (SpectraMax iD3, Molecular Devices Co., Ltd. Shanghai, China).

### Pathological Evaluation of Brain Tissue in Rats

Hematoxylin and eosin (H&E) staining was performed as previously described (Li et al., 2020). The brain specimens were fixed in 4% formalin, dehydrated, and embedded in paraffin wax. Then, coronal brain sections were cut into 5-μm slices. The sections were de-waxed with xylene, washed with water for 20 min, and then stained with hematoxylin and eosin. And the images were recorded at 200× and 400× magnification by using a DM1000 (Leica, Germany) microscopic imaging system with an optical microscope (CX21FS1, Olympus Corporation, Japan).

### Immunofluorescence Assay

Immunofluorescence was performed as previously described (Mao et al., 2020). Briefly, the de-waxed hippocampus sections were repaired by EDTA antigen retrieval solution. After blocking with 5% BSA for 30 min, the slices were incubated overnight at 4°C with primary antibodies against Mapk10, RASGRF1, RASA3, Ras, and IGF-IR in a dilution of 1:100. On the next day, the slices were incubated with Cy3-conjugated goat anti-rabbit IgG antibody for 1 h at 37°C. The nucleus was counterstained with DAPI. Simultaneously, the images were captured by a fluorescence microscope (NIKON ECLIPSE C1, Nikon Corporation, Tokyo, Japan). The immunoreactivity density was analyzed using Image-Pro Plus 6.0 software (Media cybernetics, Inc., Rockville, MD, United States).

### Acute Hypobaric Hypoxia Experiment

Forty-eight male BALB/c mice (20 ± 20 g, 6–8 weeks) were randomly assigned to six experimental groups: control group, model group, HOL group (3.3 ml/kg), DXK-H group (3.6 g/kg), DXK-M group (1.8 g/kg), and DXK-L group (0.9 g/kg). After consecutive 7 days of administration, HH-induced brain injury of mice was established according to the previous studies (Li et al., 2020).

### Western Blot Analysis

The brain tissues of animal subject to CHH and AHH insults were collected for Western blot analysis (Hou et al., 2020). The total protein of the brain tissue was extracted by a total protein extraction kit, and the total protein concentration was measured by a BCA protein quantitative kit. The proteins were separated by SDS-PAGE, with 54 μg protein loaded in each well, and transferred to a 0.45-μm PVDF membrane. The membranes were blocked with 5% BSA for 1.5 h at room temperature and subsequently incubated overnight at 4°C with primary antibodies for mice p-ERK/ERK, p-JNK/JNK, and p-p38/p38, and for rats Mapk10, RASGRF1, RASA3, Ras, and IGF-IR. Membranes were washed with TBST and subsequently incubated with anti-rabbit immunoglobulin G antibody for 2 h at room temperature. Then, ultra-signal ECL chemiluminescent solution was used to visualize the peroxidase-coated bands, and images were captured using a Chemidoc XRS Imaging System (Bio-Rad Laboratories, Inc., Hercules, CA, United States). The signal intensities of the bands of interest were quantified and normalized to β-actin using Image-Pro Plus 6.0 (Media cybernetics, Inc., Rockville, MD, United States).

### Statistical Analysis

The data were expressed as mean ± standard deviation (SD). Data analyses were processed by GraphPad Prism 6 (GraphPad software, La Jolla, United States) with one-way analysis of variance with Tukey’s post-tests. *p*-values < 0.05 were considered statistically significant.

## Result

### Identification of Main Bioactive Compounds in Duoxuekang by UPLC–Q-TOF/MS Analysis

**FIGURE 1:**
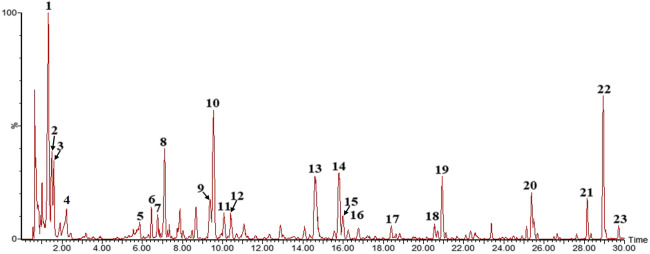
Total ion chromatogram of DXK by UPLC–Q-TOF-MS.

UPLC–Q-TOF/MS analysis was conducted to investigate the chemical profiles of DXK. Figure 1 shows the exact base peak chromatogram of DXK. Twenty-three chemical compounds are identified under the negative mode, through fitting calculation of the corresponding molecular weight on the excimer ion peak ([M-H]-) compared with the standards (Table 1). Gallic acid, ellagic acid, corilagin, rhodioloside, isorhamnetin, and rutin were identified as the preeminent bioactive compounds in DXK.

**TABLE 1:** Twenty-three chemical constituents identified by UHPLC–Q-TOF/MS.

No	RT (min)	Formula	Molecular weight	ppm	Compounds	Sources
1	1.32	C_13_H_16_O_10_	331.0665	0	1-O-glucogallin	*R. crenulata* and *P. emblica*
2	1.5	C_7_H_6_O_5_	169.0134	−1.8	Gallic acid	*P. emblica* and *R. crenulat*
3	1.59	C_13_H_12_O_11_	343.0294	−2	Mucic acid 1,4-lactone 3-O-gallate	*P. emblica*
4	2.22	C_13_H_12_O_11_	343.0294	−2	Mucic acid 1,4-lactone 5-O-gallate	*P. emblica*
5	5.86	C_20_H_20_O_14_	483.0781	−1	3,6-Digalloyl glucose	*P. emblica*
6	6.45	C_14_H_20_O_7_	299.113	−0.3	Salidroside	*R. crenulata*
7	6.77	C_14_H_10_O_9_	321.0251	1.2	Digallate	*P. emblica*
8	7.12	C_20_H_19_O_14_	483.0773	−0.4	1,6-Di-O-galloyl-glucose	*P. emblica*
9	9.38	C_41_H_28_O_10_	951.0756	1.7	Hippophaenin A	*H. rhamnoides*
10	9.55	C_27_H_22_O_18_	633.075	3.5	Corilagin	*P. emblica*
11	10.07	C_20_H_16_O_13_	463.052	1.5	Ellagic acid hexose	*P. emblica*
12	10.4	C_9_H_10_O_5_	197.0445	−2.5	Progallin A	*P. emblica*
13	14.59	C_14_H_6_O_8_	300.9982	−0.7	Ellagic acid	*P. emblica* and *H. rhamnoides*
14	15.8	C_41_H_30_O_27_	953.0901	0.5	Chebulagic acid	*P. emblica*
15	15.99	C_34_H_28_O_22_	787.1013	2.4	1,2,3,6-tetra-O-galloylglucose	*P. emblica*
16	16.25	C_34_H_26_O_22_	785.0844	0.9	Tercatain	*P. emblica*
17	18.4	C_21_H_38_O_11_	465.2334	−0.4	Rhodioloside	*R. crenulat*
18	20.54	C_21_H_20_O_11_	447.0924	−0.7	Quercetin-3-O-rhamnoside	*H. rhamnoides*
19	20.94	C_41_H_32_O_26_	939.1094	−1.1	Pentagalloyglucose	*P. emblica*
20	25.39	C_27_H_30_O_16_	609.1457	0.2	Rutin	*H. rhamnoides*
21	28.14	C_17_H_26_O_6_S	357.1365	−2	6-Gingesulfonic acid	Z*. officinale*
22	28.94	C_19_H_36_O_10_	423.2238	1.9	Rhodiooctanoside	*R. crenulat*
23	29.71	C_16_H_12_O_7_	315.0511	1.9	Isorhamnetin	*H. rhamnoides*

### Effect of Duoxuekang on Anti-Hypoxic and Anti-Fatigue in Mice

**FIGURE 2:**
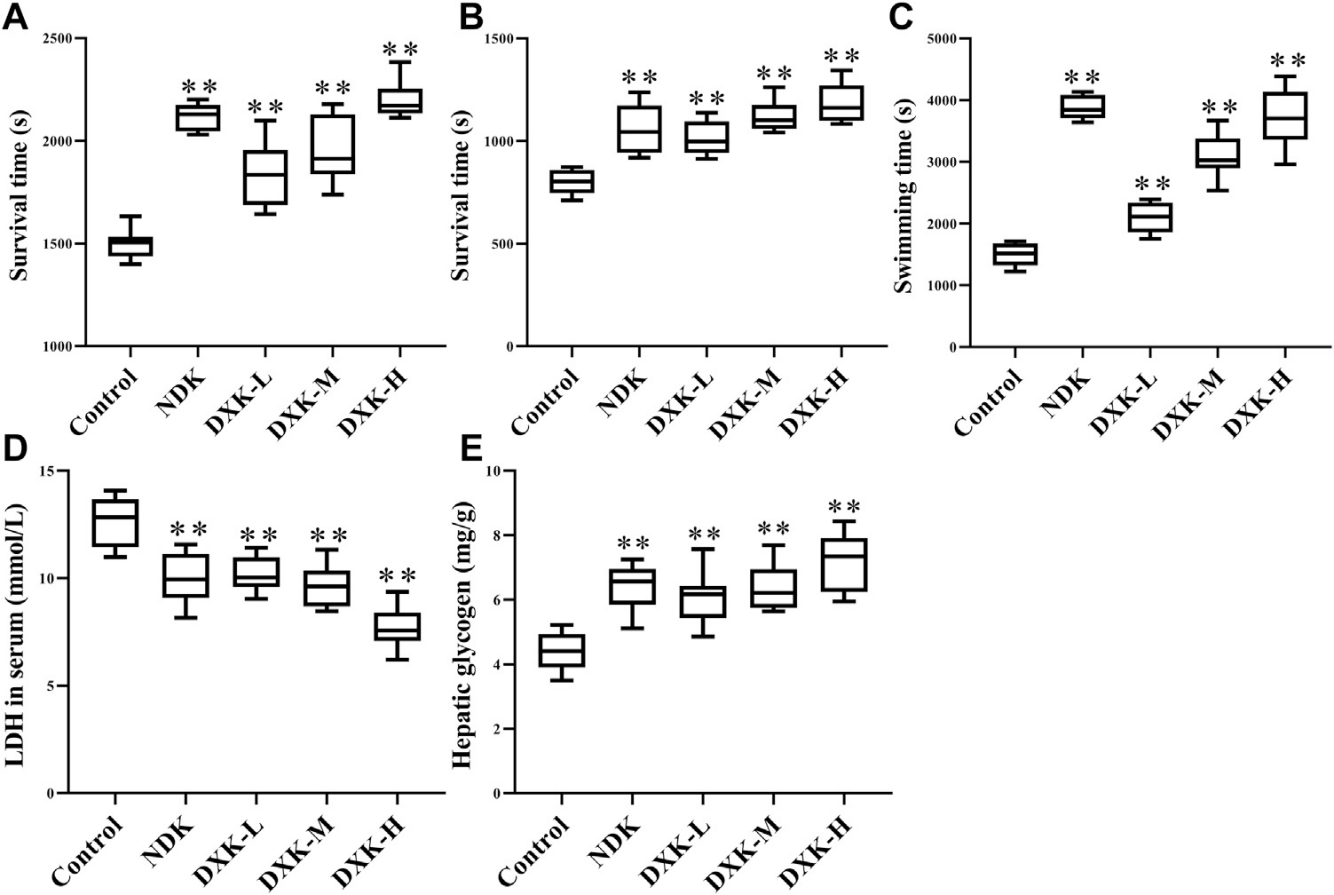
The effect of DXK on anti-hypoxia and anti-fatigue effects of mice. **(A)** The survival time of mice in normobaric hypoxia test, **(B)** the survival time of mice in sodium nitrite toxicosis test, **(C)** the weight-loaded swimming endurance time of mice, the levels of LDH in mice serum **(D),** and hepatic glycogen **(E)** after the weight-loaded swimming test. Data were expressed as the mean ± SD (*n* = 8). **p < 0.01 vs*.* the control group. DXK, Duoxuekang capsule; LDH, lactic dehydrogenase; NDK, Nuodikang capsule.

Anti-hypoxic effects of DXK on neurobehavioral impairments were examined using normobaric hypoxia test and sodium nitrite toxicosis test (Figure 2). As shown in Figure 2A,B, the survival time of mice in DXK was dose-dependently increased, compared with the control group (*P*< 0.01). In addition, the weight-loaded swimming test was used to examine the anti-fatigue effect of DXK. The weight-loaded swimming time (Figure 2C) of mice was dramatically prolonged (*p* < 0.01), LDH level (Figure 2D) was decreased (*p* < 0.01), and hepatic glycogen level (Figure 2E) was increased (*p* < 0.01) in DXK. These data suggest that DXK possessed anti-hypoxic and anti-fatigue ability.

### Effect of Duoxuekang on Hematological Parameters of Rats

**FIGURE 3:**
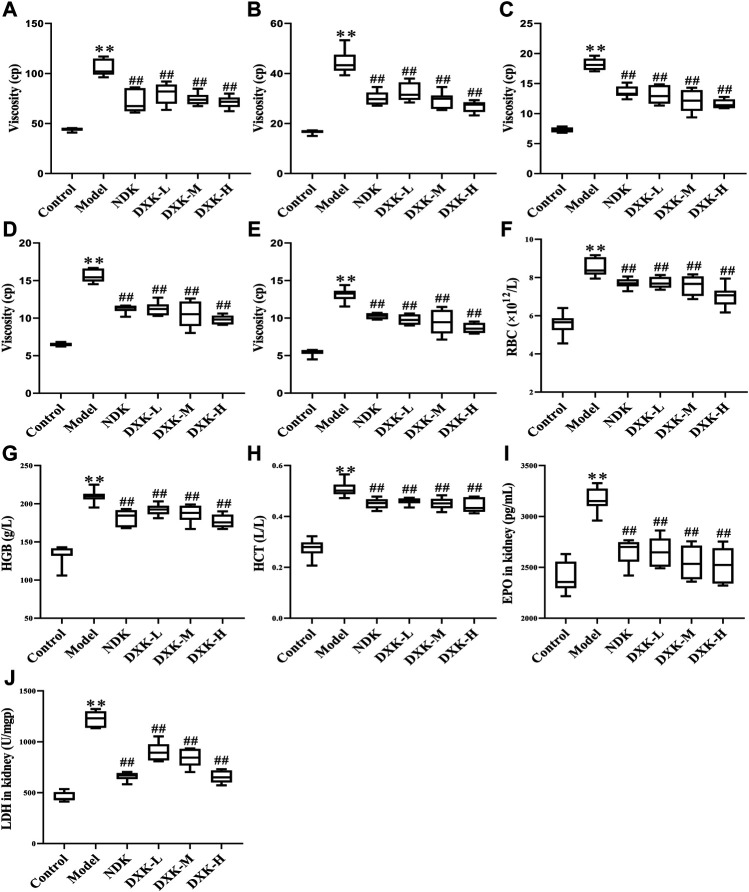
The effect of DXK on blood rheological properties, hematological parameters, and EPO and LDH of the kidney in rats with brain injury induced by CHH. The levels of the whole blood viscosity of 1 s^−1^ **(A)**, 5 s^−1^ **(B)**, 50 s^−1^ **(C)**, 100 s^−1^ **(D)**, 200 s^−1^ **(E)**, RBC **(F)**, HGB **(G)**, and HCT **(H)** of rats. The levels of EPO **(I)** and LDH **(J)** in the kidney. Data were expressed as the mean ± SD (*n* = 6). **p < 0.01 vs. the control group; *p* < 0.01 vs. the model group. DXK, Duoxuekang capsule; EPO, erythropoietin; HGB, hemoglobin; HCT, hematocrit; LDH, lactic dehydrogenase; NDK, Nuodikang capsule; RBC, red blood corpuscles.

To examine the whole blood viscosity, hematological parameters and levels of EPO and LDH in the kidney were examined after DXK treatment in the HH-induced brain injury model of rats. HH can cause an increase of the whole blood viscosity (Figures 3A–E), levels of RBC, HGB, and HCT (Figures 3F–H), and levels of EPO and LDH (Figures 3I,J) in rats. After administration of DXK, whole blood viscosity and levels of RBC, HGB, HCT, EPO, and LDH were markedly decreased (*p* < 0.01). These findings imply that DXK can reduce the increase of hematological parameters of rats caused by HH.

### Duoxuekang Ameliorates Hypobaric Hypoxia–Induced Brain Injury of Rats

**FIGURE 4:**
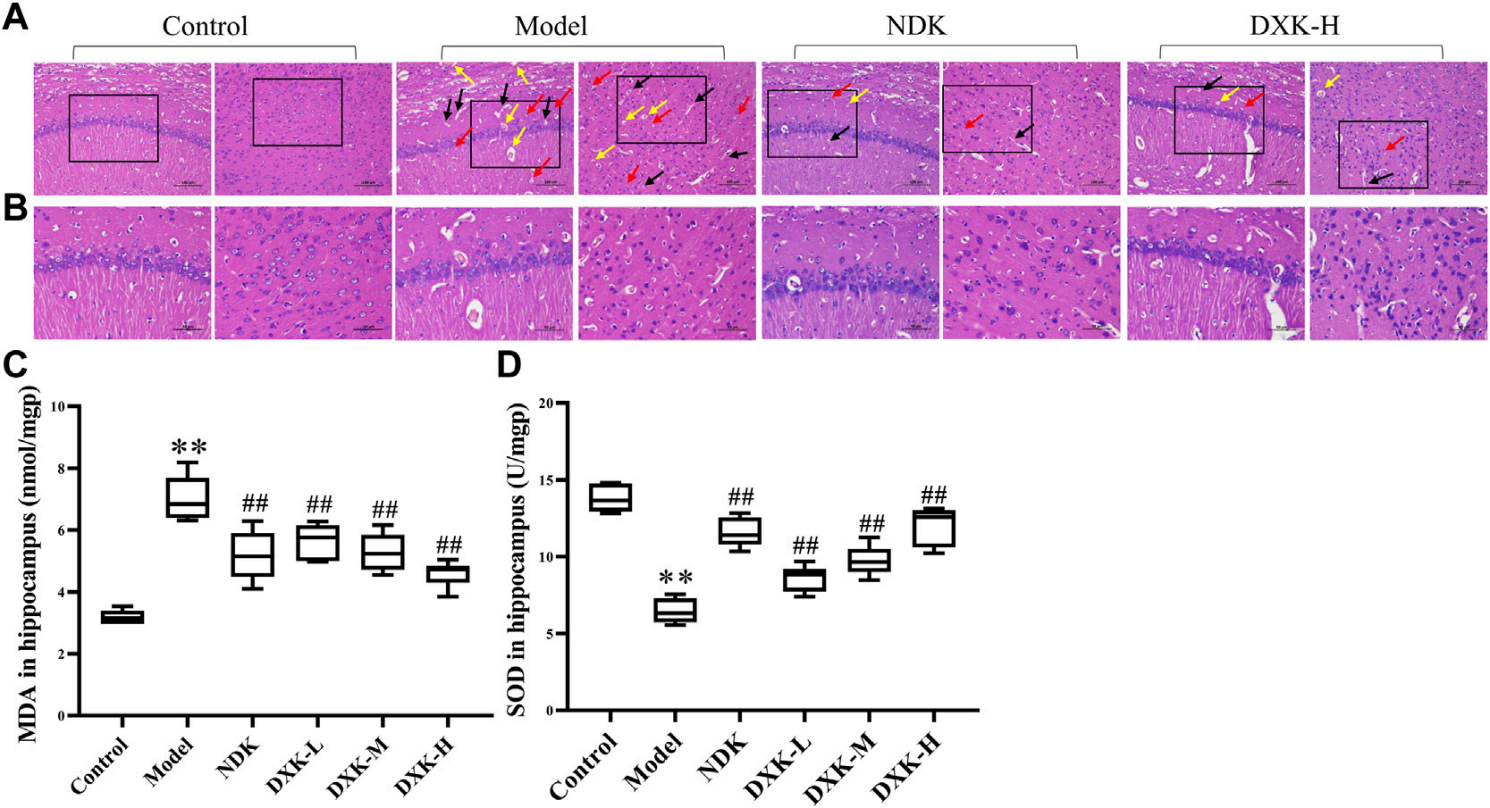
**|** The effect of DXK on brain pathomorphology and oxidative stress indexes in the hippocampus of rats with brain injury induced by CHH. **(A, B)** Representative images of H&E staining of the hippocampus (left) and cerebral cortex (right) of rats in each group (200×, scale bar: 100 μm; 400×, scale bar: 50 μm). Condensed neurons and deep-stained nucleus (red arrows), perivascular edema (black arrows), and dilation of blood vessel congestive (yellow arrows). The levels of MDA **(C)** and SOD **(D)** in the hippocampus. Data were expressed as the mean ± SD (*n* = 6). **p < 0.01 vs. the control group; ^##^ *p* < 0.01 vs. the model group. DXK, Duoxuekang capsule; MDA, malondialdehyde; NDK, Nuodikang capsule; SOD, superoxide dismutase.

We observed the effects of DXK administration on the degree of brain injury after HH exposure by using H&E. The H&E staining representative images of the hippocampus and cerebral cortex of the rats showed that HH causes shrinking of neurons with darkly stained pyknotic nuclei, disordered array of neurons, perivascular edema, and vascular dilatation and congestion (Figures 4A,B). After administration of DXK, these pathological changes were significantly improved. In addition, compared with the control group, the level of MDA (Figure 4C) was increased, while SOD (Figure 4D) was decreased after HH exposure. However, after administration of DXK, MDA was signally decreased, and SOD was increased. In particular, DXK caused the changes of oxidative stress biomarkers in a dose-dependent way. All in all, the results above indicated that DXK has a good cerebral protective effect in the HH-induced brain injury model.

### Duoxuekang Regulates RAS and Mitogen‑Activated Protein Kinase Signaling Pathways

**FIGURE 5:**
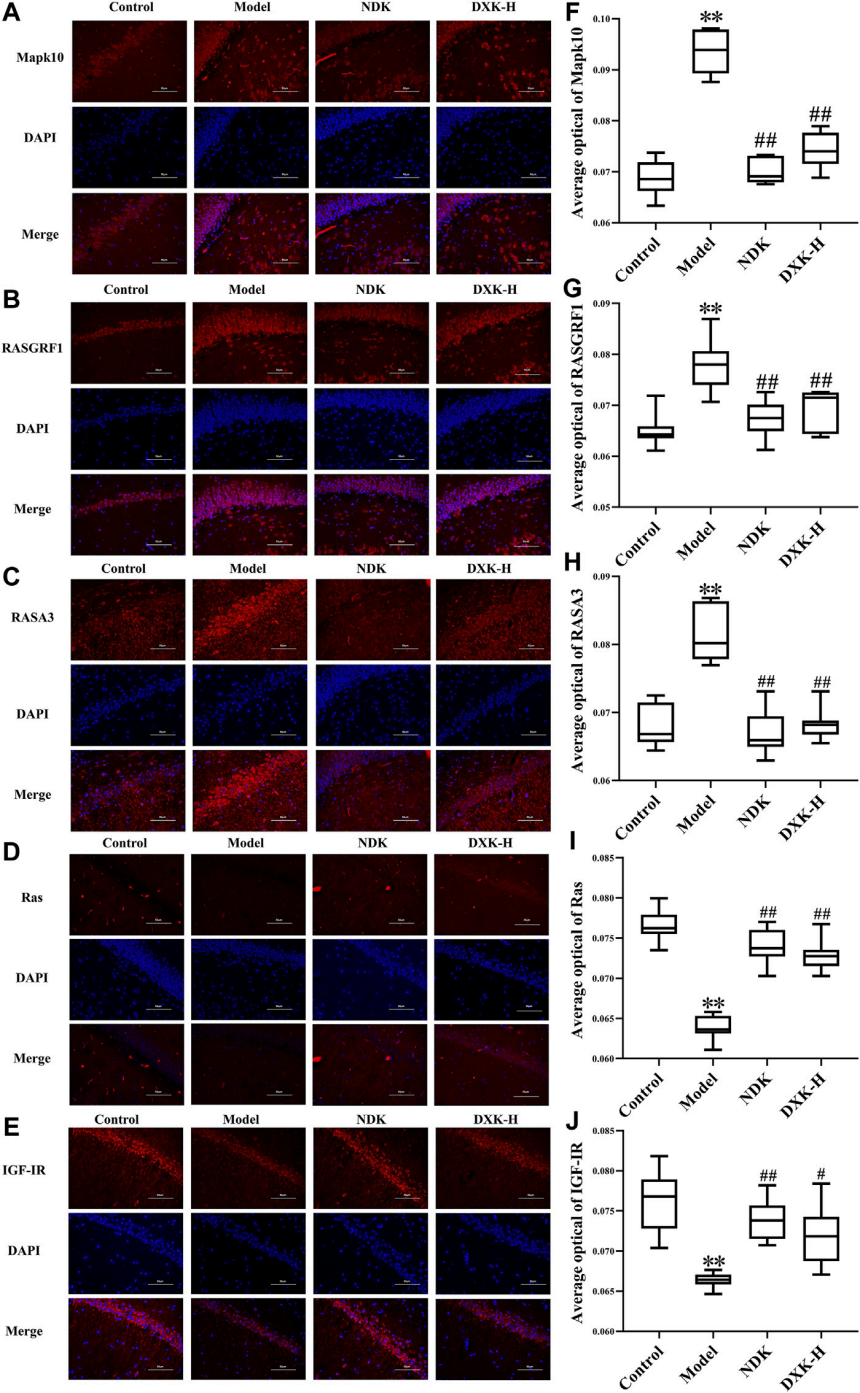
The effect of DXK on the protein levels of Mapk10, RASGRF1, RASA3, Ras, and IGF-IR in the hippocampus of rats with brain injury induced by CHH. Representative microphotographs of immunofluorescence staining (400×) for identification of Mapk10 **(A)**, RASGRF1 **(B)**, RASA3 **(C)**, Ras **(D)**, and IGF-IR **(E)** (red color); average optical of Mapk10 **(F)**, RASGRF1 **(G)**, RASA3 **(H)**, Ras **(I)**, and IGF-IR **(J)**. Data were expressed as the mean ± SD (*n* = 6). **p < 0.01 vs. the control group; ^#^ *p* < 0.05, ^##^ *p* < 0.01 vs. the model group. DXK, Duoxuekang; NDK, Nuodikang capsule.

**FIGURE 6:**
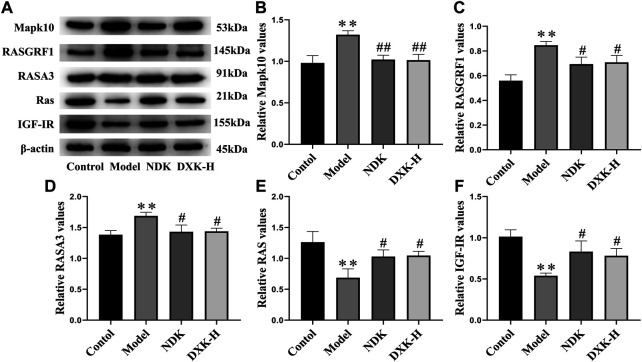
The effect of DXK on the protein levels of signaling pathway–related proteins in the hippocampus of rats with brain injury induced by CHH. **(A)** Representative protein bands of Mapk10, RASGRF1, RASA3, Ras, and IGF-IR. **(B–F)** The corresponding quantitative statistical results of Mapk10 **(B)**, RASGRF1 **(C)**, RASA3 **(D)**, Ras **(E)**, and IGF-IR **(F)**. Data were expressed as the mean ± SD (*n* = 3). **p < 0.01 vs. the control group; ^#^ *p* < 0.05, ^##^ *p* < 0.01 vs. the model group. DXK, Duoxuekang; NDK, Nuodikang capsule.

**FIGURE 7:**
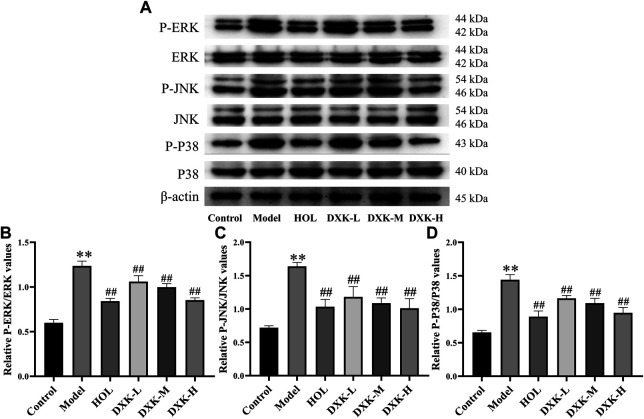
The effect of DXK on the expression of the MAPK signaling pathway–related proteins in the cerebral cortex of mice with brain injury induced by AHH. **(A)** Representative protein bands of p-ERK, ERK, p-JNK, JNK, p-p38, and p38. The corresponding quantitative statistical results of p-ERK/ERK **(B)**, p-JNK/JNK **(C)**, and p-p38/p38 **(D)**. Data were expressed as the mean ± SD (*n* = 3). **p < 0.01 vs. the control group; ^##^ *p* < 0.01 vs. the model group. DXK, Duoxuekang; HOL, Hongjingtian oral liquid.

RAS and MAPK signaling pathways were closely related to brain injury after HH exposure (Xu et al., 2016; Wang et al., 2018). Thus, we evaluated whether DXK influenced RAS and MAPK signaling pathways to alleviate HH-induced brain injury. Immunofluorescence (Figure 5) and Western blot (Figure 6) were performed to assess the expression of RAS signaling pathways in rats’ hippocampus. Compared with the control group, HH exposure increased Mapk10, RASGRF1, and RASA3 (*p* < 0.01), as well as decreased Ras and IGF-IR (*p* < 0.01), while DXK treatment reversed the tendency. Simultaneously, the result of Western blot showed that HH exposure substantially increased phosphorylation of ERK, JNK, and p38 in the mice cerebral cortex, and there was a significant difference following treatment with DXK (Figure 7, *p* < 0.01). The therapeutic effect of Duoxuekang in HH-induced brain injury is mainly through the regulation of RAS and MAPK pathways.

## Discussion

As the unique HH environment in the plateau, the imbalance of oxygen supplies and consumption of human body can lead to excessive reactive oxygen species production and oxidative stress injury (Farías et al., 2016; Badran et al., 2019). The brain injury caused by HH can cause a decrease in learning, memory, and the ability to deal with complexity (Taylor et al., 2016). Furthermore, long-term HH environment triggered the erythrocytosis, high-blood viscosity, and the decline of oxygen uptake and transportation (Lu et al., 2017). It has been verified that HH caused edematous neurons, enlarged perivascular space, and shrinking of neurons with darkly stained pyknotic nuclei (Wang et al., 2018; Wang et al., 2019). Tibetan medicine, as a traditional plateau medical system recognized by the world, has a unique systematic theory and practical experience in the prevention and treatment of altitude sickness. In theory of Tibetan medicine, DXK can mainly treat Chiba disease by promoting blood circulation, removing blood stasis, and clearing heat and detoxification (Ga et al., 2019). Previous studies have found that DXK can improve HAPC and HH-induced brain injury (Li et al., 2020). However, the pharmacodynamic material basis of DXK against HH-induced brain injury is not clear. In this study, a total of 23 different kinds of compounds of DXK were identified using UPLC–Q-TOF/MS (Figure 1 and Table 1). Synchronously, there was evidence that gallic acid (from *P. emblica* and *R. crenulate*) (Sun et al., 2014), ellagic acid (from *P. emblica* and *H. rhamnoides*) (Dhingra et al., 2017), salidroside (derived from *R. crenulate*) (Fan et al., 2020), as well as isorhamnetin (Gong et al., 2020) and rutin (Sundaram et al., 2018) (both from *H. rhamnoides*) had antioxidative stress, anti-inflammation, and neuroprotective effects.

The normobaric hypoxia test and the sodium nitrite toxicosis test were implemented for the evaluation of DXK antioxidant activity (Cui et al., 2018). The presented experiments uncovered that DXK had significant antioxidant activity which can prolong the survival time of mice in both tests. The weight-loaded swimming test was generally noted to assess the anti-fatigue activity of drugs (Yang et al., 2020). Besides, intense exercise can cause a decrease of hepatic glycogen and an increase of LDH levels in blood (Zhang et al., 2014; Xie et al., 2020). Our results indicated that DXK can prolong the exhaustion time on the weight-loaded swimming test by decreasing the LDH activity and increasing the hepatic glycogen level. These results indicate that DXK possessed anti-hypoxic and anti-fatigue ability.

According to previous studies, the HAPC model in rats was successfully established by evaluating their RBC, HGB, HCT, and the whole blood viscosity (Cowan et al., 2012; Kim et al., 2019). Hypoxia stimulated a persistent increase in EPO mainly generated by the kidney, causing an increase of RBC in response to changes in blood oxygen availability, ultimately leading to HAPC (Zhou et al., 2012; Bunn, 2013). It is confirmed that hypoxia inducible factor-1α (HIF-1α) gene and protein expression are increased after HH exposure (Xie et al., 2017; Li et al., 2021). In addition, HIF-1α can promote the expression of EPO, which promotes the maturation of RBC (Marzo et al., 2008). The overexpression of EPO can lead to the increase of RBC, which results in an increase in blood viscosity (Ogunshola et al., 2006). In previous studies, we established the HAPC animal model in SD rats and confirmed that HH can cause the increase of HIF-1α expression in the hippocampus and cortex, while repression after DXK treatment (Wu et al., 2009). In this study, we observed that DXK can reduce the increase of blood viscosity and EPO caused by HH. Results above indicate that DXK can decrease the blood viscosity by reducing the expression of EPO. Studies have found that HH can cause cerebral blood–brain barrier (BBB) dysfunction, increased vascular permeability, as well as aggravated hippocampal and cortical damage (Wilson et al., 2009; Liu et al., 2015; Coimbra-Costa et al., 2017). As a crucial promoter of apoptosis, oxidative stress indexes of MDA and SOD contributed to increased dead neuronal cell (Wu et al., 2019). In this study, we found that DXK can improve the damage of cortex and hippocampus induced by HH, decrease MDA, and increase the SOD level in rats. Our study showed that DXK can ameliorate HH-induced brain injury and oxidative stress.

**FIGURE 8:**
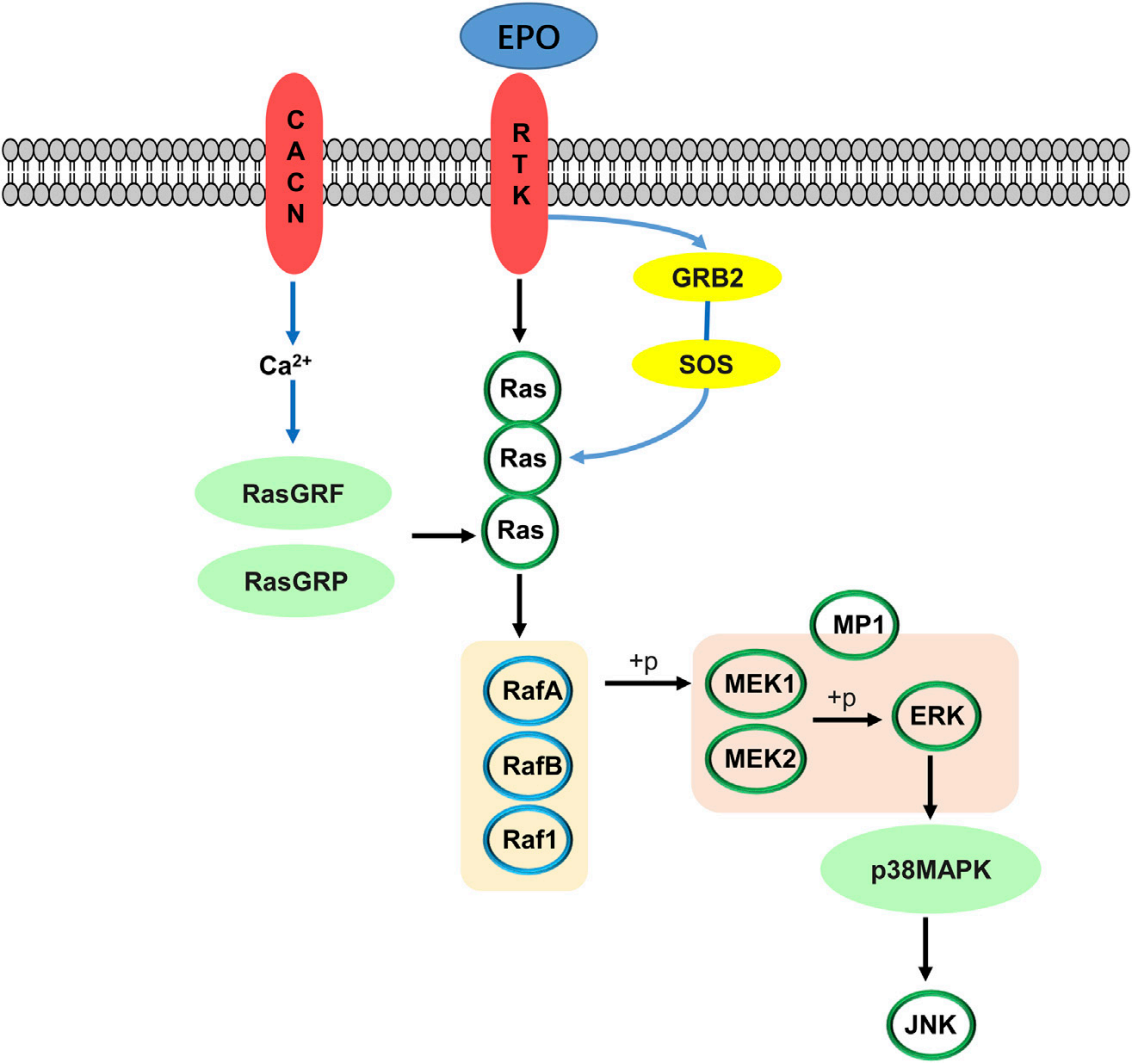
Mechanism of activation of the RAS/MAPK signaling pathway by erythropoietin.

Clinical studies have confirmed the positive effects of NDK and HOL in the treatment of brain injury caused by HH (Meng et al., 2015; He and Zhang, 2017). Therefore, we selected NDK and HOL as positive drugs in brain injury models of AHH and CHH, respectively. In addition, Mapk10 was selectively expressed in the central nervous system (CNS), and the lack of Mapk10 conferred neuroprotection (Pirianov et al., 2007; Wen et al., 2016). It is confirmed that activation of RAS and MAPK signaling pathways is associated with EPO, and its mechanism is shown in Figure 8. (Miura et al., 1994; Marzo et al., 2008). Besides, RASGRF1, observed in mature neurons of the hippocampus (Zhu et al., 2013), was a neuron-specific guanine nucleotide exchange factor for Ras proteins (Tonini et al., 2001), mediating the activation of oxidative stress by regulating Ras family proteins (Tsai et al., 2018). Furthermore, RASA3, acting as a suppressor of Ras function (Hancock, 2003), was critical for the accommodation of platelet adhesion (Stefanini and Bergmeier, 2016). In addition, IGF-I, locally produced by neurons and glial cells, exerted significant neuroprotection during acute brain injury insult (De Magalhaes Filho et al., 2017). Synchronously, IGF-IR was widely expressed in CNS, producing IGF-I and IGF-binding proteins, and its activation mediated neuroprotective effects under hypoxia condition (Garcia-Segura et al., 2006). In this study, HH induced increases of Mapk10, RASGRF1, and RASA3, as well as decreases of Ras and IGF-IR, while DXK treatment conversed the tendency. These results indicate that the regulation of the RAS pathway is related to the cerebral protective effect of DXK. In addition, oxidative stress motivated the MAPK signaling pathway, leading to cellular damage (Liu et al., 2018). In our previous study, we established the HH-induced brain injury model in BALB/c mice and found that HH can lead to oxidative damage of the brain (Li et al., 2020). DXK can significantly improve oxidative stress injury of the brain induced by HH (Li et al., 2020). In our study, the phosphorylation of ERK, JNK, and p38 was activated by HH, while causing repression after DXK treatment, suggesting that DXK can effectively antagonize HH-induced oxidative stress injury and activation of the MAPK signaling pathway.

In conclusion, our results show that DXK has cerebral protection effect against HH through the decrease of the whole blood viscosity and reduction in oxidative damage, *via* regulating RAS and MAPK signaling pathways. However, there are some limitations in this study. First, we have not absolutely confirmed the potential constituents in DXK penetrating the BBB to exert cerebral protection. Second, although we have proved that DXK can regulate RAS and MAPK signaling pathways, the interactions between them still need to be further investigated. In the following experiments, we will clarify the pharmacodynamic material basis of DXK hypoxic brain protection by imaging mass spectrometry and microfluidic BBB chips (Wang et al., 2020). Meanwhile, we will elucidate the interactions between RAS and MAPK signaling pathways through gene silencing and protein expression inhibitors.

## Data Availability

The original contributions presented in the study are included in the article/Supplementary Material; further inquiries can be directed to the corresponding authors.
